# Role of monoamine-oxidase-A-gene variation in the development of glioblastoma in males: a case control study

**DOI:** 10.1007/s11060-019-03294-w

**Published:** 2019-09-25

**Authors:** Rickard L. Sjöberg, Wendy Yi-Ying Wu, Anna M. Dahlin, Spiridon Tsavachidis, The Gliogene Group, Margaret R. Wrensch, Sara H. Olson, Michael E. Scheurer, Dora Il’yasova, Daniel Lachance, Georgina N. Armstrong, Lucie S. McCoy, Ching C. Lau, Elizabeth B. Claus, Jill S. Barnholtz-Sloan, Joellen Schildkraut, Francis Ali-Osman, Siegal Sadetzki, Christoffer Johansen, Richard S. Houlston, Robert B. Jenkins, Jonine L. Bernstein, Ryan T. Merrell, Faith G. Davis, Rose Lai, Sanjay Shete, Christopher I. Amos, Beatrice S. Melin, Melissa L. Bondy, Melissa L. Bondy, Beatrice Melin

**Affiliations:** 1 Department of Clinical Science, Neuroscience, Umeå University, Umeå, Sweden; 2 Department of Neurosurgery, University Hospital of Northern Sweden, 901 85 Umeå, Sweden; 3 Department of Radiation Sciences, Oncology, Umeå University, Umeå, Sweden; 4 Department of Medicine, Section of Epidemiology and Population Sciences, Dan L. Duncan Comprehensive Cancer Center, Baylor College of Medicine, Houston, TX USA

**Keywords:** MAOA genetics glioblastoma males

## Abstract

**Background:**

The Mono-amine oxidase-A (MAO-A) enzyme is involved in the degradation and regulation of catecholamines such as serotonin, dopamine, epinephrine and nor-epinephrine. Preclinical studies suggest that this enzyme may contribute to an environment favorable for growth of malignant glioma. The *MAO-A* gene is located on the X-chromosome and has at least one functional genetic polymorphism. The aim of the present study was to explore possible effects of *MAO-A* genotype on development of glioblastoma in males.

**Methods:**

Genotypes for 437 glioma cases and 876 population-based controls from the Swedish Glioma International Case–Control study (GICC) were compared. We analyzed the germline DNA using the Illumina Oncoarray. We selected seven single nucleotide polymorphisms (SNPs) located in the *MAO-A* gene, and imputed genotypes based on data from the 1000 genomes project. We used 1579 male glioblastoma cases and 1875 controls comprising the whole GICC cohort for subsequent validation of findings.

**Results:**

The rs144551722 SNP was a significant predictor of development of glioblastoma in males (p-value = 0.0056) but not in females even after correction for multiple testing. We conducted haplotype analysis to confirm an association between *MAO-A* gene and risk of glioblastoma (p-value = 0.016). We found similar results in the validation sample.

**Conclusions:**

These results suggest the possibility of a role for the MAO-A enzyme and the *MAO-A* gene in the development of glioblastoma in males.

## Introduction

The risk for developing glioma is roughly 1.4 times greater for males than females [1, 2]. We have recently described 25 genetic variants associated with development of glioma in both genders using a genome wide approach [3]. In addition, a recent study using a similar approach but focusing on the X-chromosome identified four regions of potential interest that remained statistically significant after correction for 250,000 significance tests [4]. In these studies, an agnostic, exploratory methodology was applied, according to which all available polymorphisms were analyzed, regardless of known function.

However, a potential useful alternative strategy in the study of genetically determined sex differences is the candidate gene study. In this context such a strategy might mean focusing on known x-linked genetic polymorphisms that, for theoretical reasons, might be expected to be related to glioma development.

One such candidate is the *MAO-A-*gene that is located on the X-chromosome (Xp 11.23) [5]. The gene codes for a protein that is involved in the degradation of several neurotransmitters most important serotonin, dopamine, epinephrine and nor-epinephrine in a tissue specific manner. Functional variations in the *MAO-A-*gene have in several studies been associated with behavioral outcomes particularly in males. One early example is a study of a Dutch family, in which a stop codon in the gene, associated with severe antisocial behavior in males, was identified [6]. More recently much interest has been focused on a functional variable number of tandem repeat (VNTR-)polymorphism in the promoter region of the *MAO-A* gene commonly referred to as the *MAOA*-Linked Polymorphic Region (*MAOA*-LPR) [7, 8]. The low function variant of this VNTR has in several studies been linked to behavioral and neurophysiological phenotypes in males, [9–14] and to some lesser extent females [15, 16]. This has led to a common assumption within the field of behavioral genetics that the polymorphism may be important in the overall regulation of monoaminergic systems [17].

From a theoretical point-of-view there are at least three possible mechanisms by which variation in the *MAOA-*gene may be relevant to the development of glioma. The two first of these would be through the regulation of monoaminergic neurotransmission, particularly Serotonin and Dopamine [18].

### Regulation of stem cell proliferation

It has been reported that both Dopamine and Serotonin influence proliferation of neural progenitor cells in the sub ventricular zone, [19–21] and the dentate gyrus [22], respectively. Converging evidence suggests that the cells that give rise to glioma development may share many important features with these progenitor cells [23, 24] suggesting the possibility that Dopamine and Serotonin may influence cell proliferation in glioma precursor cells as well. This would provide potential for functional genetic variation in a gene relevant to the regulation of these neurotransmitters to influence early glioma development.

### Regulation of angiogenesis

Particularly Dopamine has in several studies been implicated as an inhibitor of angiogenesis through interaction with the Vascular endothelial growth factor (VEGF) pathway [25, 26]. Since angiogenesis is a key feature of glioma development, particularly in glioblastoma, a genetic polymorphism with the potential to regulate levels of an endogenous inhibitor of angiogenesis may also be a possible candidate gene.

### Effects of oxidative stress

Findings of increased serum levels of reactive oxygen species (ROS) in patients who later develop Glioblastoma suggests a role for oxidative stress in the genesis of this disease [27]. Levels of oxidative stress including increased ROS has been associated with MAO-A over-expression in prostate cancer models [28, 29]. Therefore, it would seem to make sense that a functional polymorphism that regulates MAO-A transcription could influence levels of oxidative stress in a way that might influence glioma development.

More direct experimental evidence for a role of the MAO-A enzyme in glioma development was recently provided in experiments reported by Kushal et al. [30]. These authors found increased levels of the MAO-A protein in glioma tissue. Furthermore, they found that inhibition of MAO-A activity was cytotoxic to glioma cells in-vitro and that it reduced proliferation, microvessel density, and invasion of glioma tissue in a rat model.

In line with the reasoning presented above, the purpose of the present study was to investigate the specific hypothesis that variation in the *MAOA* gene is associated with development of glioblastoma in males. We investigated this hypothesis using a case–control approach.

## Methods

### Swedish sample

The study subjects included in the risk analysis were those who participated in the Swedish Glioma International Case–Control (GICC) study. Details of patient recruitment, data collection, and quality control are available in previous publications [3, 31]. In brief, cases were between the ages of 18–80 years, and recruited between the years 2010 and 2013 from five hospitals in Sweden. In total, 472 histologically confirmed newly diagnosed glioma cases and 908 population-based controls were genotyped. We excluded subjects with < 99% sample genotyping call-rate, subjects with inconsistencies between reported sex and sex estimated by genotype, subjects with < 80% estimated European ancestry, subjects identified as outliers in principle component analyses and one of each pair of individuals with spurious relations (PI-HAT > 0.2). We also excluded cases with rare glioma diagnoses (SNOMED codes 93913, 93923, 94121, 94211, 94423, and 95051). After this quality control, 437 cases and 876 controls were included. There were 175 male and 94 female GBM cases.

### SNP selection

We selected seven SNPs in the *MAO-A* gene. Because MAO-LPR was not directly sequenced in our study, we used Haploview (version 4.2) to select 3 SNPs with minor allele frequency > 0.1 that tag variation in the region of the MAO-LPR. For this purpose, we used reference data (± 10 kb from MAO-LPR) from the 1000 genomes projects (phase 3, European population) [32]. Details of the analyzed SNPs are shown in Table 1.

**Table 1:** Analyzed SNPs in the *MAO-A* gene

SNP numbers	Location	Most severe consequence	Alleles	Info	Certainty
rs5905513	43491842	Intergenic variant	G/A	0.837	0.925
rs144551722	43491877	Intergenic variant	G/A	0.784	0.948
rs5906260	43498619	Intergenic variant	C/T	0.998	0.999
rs1465108	43538209	Intron variant	A/G	0.995	0.998
rs909525	43553202	Intron variant	C/T	0.978	0.991
rs979605	43601363	Intron variant	A/G	0.999	1
rs2239448	43602679	Intron variant	T/C	0.999	1

### Genotyping and imputation

We used the Illumina Oncoarray to genotype the SNPs. We imputed untyped variants in the *MAOA* gene using the IMPUTE2 and SHAPEIT2 software, and data from the 1000 genomes project as reference [33–36]. Before imputation, we excluded SNPs with poor call-rate (< 95%), p-value from Hardy–Weinberg test < 1 × 10^–6^, minor allele frequency < 0.01, and all A/T and C/G SNPs. Imputation info scores for SNPs in *MAO-A* are presented in Table 1. For imputed variants, genotypes were called based on the highest imputed genotype probability. A genotype call was set to “missing” in subjects where all three genotype probabilities for a variant were < 0.9.

### Statistical analysis

We performed gender-stratification analyses to test the associations between genotype/allele frequencies and glioma risk using chi-square test/Fisher exact test. We conducted haplotype analysis of 5 SNPs in the *MAO-A* gene, and logistic regression to estimate odds ratios (OR) and 95% confidence intervals (CI). We applied Bonferroni correction for the SNP-analysis by setting the critical *p *level to 0.00714 (005/7).

### Validation set

The cases and controls from the entire GICC study earlier presented was used for validation [31]. In total there were 2614 male glioma cases, of whom 1579 was glioblastoma. They were compared to 1875 male controls.

## Results

### Analysis of the Swedish sample

#### SNP-analysis

Table 2 describes analyses of association between the seven selected SNPs in male cases and controls, and the p-values were < 0.05 for all seven SNPs. After correction for multiple testing the rs144551722 SNP remained significant (p-value = 0.0056). There were no significant effects for lower grade gliomas or glioblastomas (as can be seem in Table 3), for females.

**Table 2:** Association of MAO-A polymorphisms in Swedish 770 males

SNP		Control (N = 516)		GBM case (N = 175)		Non GBM case (N = 79)		p Value for GBM	p Value for non GBM
		N	(%)	N	(%)	N	(%)		
rs5905513	A	211	(48.96)	84	(61.31)	28	(43.75)	0.0154	0.5198
	G	220	(51.04)	53	(38.69)	36	(56.25)		
rs144551722	G	392	(87.89)	148	(96.10)	65	(94.20)	**0.0056**	0.1807
	A	54	(12.11)	6	(3.90)	4	(5.80)		
rs5906260	T	337	(65.31)	134	(76.57)	54	(68.35)	0.0076	0.6865
	C	179	(34.69)	41	(23.43)	25	(31.65)		
rs1465108	G	333	(64.91)	133	(76.00)	53	(67.09)	0.0089	0.8017
	A	180	(35.09)	42	(24.00)	26	(32.91)		
rs909525	T	317	(62.52)	128	(73.56)	52	(66.67)	0.0108	0.5621
	C	190	(37.48)	46	(26.44)	26	(33.33)		
rs979605	G	340	(65.89)	133	(76.00)	54	(68.35)	0.0167	0.7617
	A	176	(34.11)	42	(24.00)	25	(31.65)		
rs2239448	C	339	(65.70)	133	(76.00)	54	(68.35)	0.0148	0.7363
	T	177	(34.30)	42	(24.00)	25	(31.65)		

Bold is used to highlight significant p-values after Bonferroni correction for 7 tests (p = 0.05/20 = 0.0072)

**Table 3:** Association of MAO-A polymorphisms in Swedish 530 females

SNP	Control (N = 360)		GBM Case (N = 94)		Non GBM case (N = 76)		p Value for GBM	p Value for non GBM
	N	(%)	N	(%)	N	(%)		
rs5905513								
AA	68	(28.94)	9	(16.36)	15	(30.00)	0.0889	0.2147
AG	112	(47.66)	27	(49.09)	18	(36.00)		
GG	55	(23.40)	19	(34.55)	17	(34.00)		
rs144551722								
GG	230	(79.04)	58	(86.57)	48	(87.27)	0.2769*	0.4147*
GA	58	(19.93)	8	(11.94)	7	(12.73)		
AA	3	(1.03)	1	(1.49)	0	(0.00)		
rs5906260								
TT	164	(45.81)	39	(41.94)	38	(50.00)	0.2563	0.3379
CT	161	(44.97)	40	(43.01)	28	(36.84)		
CC	33	(9.22)	14	(15.05)	10	(13.16)		
rs1465108								
GG	164	(45.81)	38	(41.30)	38	(50.00)	0.2725	0.3759
GA	160	(44.69)	40	(43.48)	28	(36.84)		
AA	34	(9.50)	14	(15.22)	10	(13.16)		
rs909525								
TT	149	(43.06)	29	(32.58)	33	(45.21)	0.1544	0.4298
TC	156	(45.09)	45	(50.56)	28	(38.36)		
CC	41	(11.85)	15	(16.85)	12	(16.44)		
rs979605								
GG	180	(50.00)	42	(44.68)	37	(48.68)	0.1812	0.8674
GA	149	(41.39)	38	(40.43)	31	(40.79)		
AA	31	(8.61)	14	(14.89)	8	(10.53)		
rs2239448								
CC	180	(50.00)	42	(44.68)	37	(48.68)	0.1812	0.8674
CT	149	(41.39)	38	(40.43)	31	(40.79)		
TT	31	(8.61)	14	(14.89)	8	(10.53)		

^*^Fisher exact test

#### Haplotype analysis

Second, a haplotype analysis including rs144551722, rs1465108, rs909525, rs979605, rs2239448 was performed in order to further investigate the relation between genotype and glioblastoma in males. The overall haplotype pattern was, as seen in Table 4, significant (p-value = 0.016).

**Table 4:** Haplotype analysis of 5 SNPs in the MAO-A gene in 154 Swedish males with glioblastoma and 441 controls

Haplotype^a^	Control		GBM		OR	95% CI	p Value
	N	(%)	N	(%)			
GGTGC	303	(70.6)	126	(29.4)	1.00		
GGT**AT**	12	(85.7)	2	(14.3)	0.40	(0.06, 1.50)	0.236
GG**C**GC	17	(77.3)	5	(22.7)	0.71	(0.23, 1.83)	0.505
G**ACAT**	56	(78.9)	15	(21.1)	0.64	(0.34, 1.15)	0.155
**AAC**GC	10	(90.9)	1	(9.1)	0.24	(0.01, 1.28)	0.176
**AACAT**	43	(89.6)	5	(10.4)	0.28	(0.10, 0.66)	0.008

^a^SNP numbers: rs144551722, rs1465108, rs909525, rs979605, rs2239448

Global p-value = 0.016

### Validation in the whole GICC cohort

#### SNP-analysis

After analyses of the Swedish sample, we conducted a validation analysis on the whole GICC cohort. As can be seen in Table 5, rs144551722, which was the only SNP to remain statistically significant after correction for multiple testing in the Swedish dataset, was significant in the whole GICC cohort as well (p < 0.05).

**Table 5:** Association of MAO-A polymorphisms in males in the whole GICC cohort

SNP	Control (N = 1875)		GBM case (N = 1579)		Non GBM Case (N = 1035)		p Value for GBM	p Value for non GBM
	N	(%)	N	(%)	N	(%)		
rs5905513								
A	1036	(44.7)	913	(57.8)	436	(42.1)	0.13	0.185
G	839	(55.3)	666	(42.2)	599	(57.9)		
rs144551722								
G	1520	(81.1)	1322	(83.7)	851	(82.2)	**0.0441**	0.455
A	355	(18.9)	257	(16.3)	184	(17.8)		
rs5906260								
T	1311	(69.9)	1122	(71.1)	740	(71.5)	0.477	0.396
C	564	(30.1)	457	(28.9)	295	(28.5)		
rs1465108								
G	1301	(69.4)	1115	(70.6)	739	(71.4)	0.434	0.272
A	574	(30.6)	464	(29.4)	296	(28.6)		
rs909525								
T	1235	(65.9)	1058	(67)	703	(67.9)	0.492	0.268
C	640	(34.1)	521	(33)	332	(32.1)		
rs979605								
G	1298	(69.2)	1105	(70)	741	(71.6)	0.656	0.19
A	577	(30.8)	474	(30)	294	(28.4)		
rs2239448								
C	1299	(69.3)	1104	(69.9)	295	(28.5)	0.711	0.22
T	576	(30.7)	475	(30.1)	740	(71.5)		

#### Haplotype analysis

As seen in Table 6 a replication of the haplotype analysis in the full GICC case control did not reach significance (p-value = 0.1).

**Table 6:** Haplotype analysis of 5 SNPs in the MAO-A gene in 2307 males with glioblastoma and 1850 controls from the whole Glioma International Case–Control (GICC) study

Haplotype^a^	Control		GBM		OR	95% CI	p Value
	N	(%)	N	(%)			
GGTGC	1169	(39.7)	1778	(60.3)	1.00		
GGT**AT**	56	(48.3)	60	(51.7)	0.70	(0.48, 1.04)	0.067
GG**C**GC	66	(41.2)	94	(58.8)	0.94	(0.67, 1.31)	0.74
G**ACAT**	210	(39.2)	326	(60.8)	1.02	(0.84, 1.24)	0.848
**AAC**GC	47	(49)	49	(51)	0.69	(0.45, 1.05)	0.072
**AACAT**	302	(42.6)	407	(57.4)	0.89	(0.75, 1.05)	0.159

^a^SNP numbers: rs144551722, rs1465108, rs909525, rs979605, rs2239448

Global p-value = 0.1

## Discussion

The purpose of the present study was to investigate the hypothesis that polymorphisms in the *MAO-A*-gene are associated with development of glioma in males. As described in the introduction there are several theoretical reasons to assume a potential involvement of the MAO-A-enzyme in glioma development. However, the direct impetus for performing the study was a recently published series of experiments demonstrating direct effects of the MAOA protein on central features of glioma development [30]. The *MAO-A*-gene is x-linked and variation in the functional *MAO-A*-LPR is known to interact with androgens in vitro and in vivo [12, 37]. Both facts make it reasonable to assume that the effects would be considerably stronger in males.

The results of the present study are generally in line with these predictions. That is one of the SNPs that was selected to tag the genetic region spanning the *MAO-LPR* (rs144551722) in the present study was significantly associated with glioblastoma in the Swedish sample. A replication study in the full GICC cohort showed weaker findings, but still confirmed an association between glioblastoma and the G/C variant of the rs144551722.

Taken as a whole our results support the hypothesis that MAOA-genotype may play a role in development of glioblastoma in males. One possible explanation for differences in the strength of results between samples may of course be that the findings are spurious. Among other possible explanations may be that some genetic or environmental risk exposures that interact with the *MAOA*-genotype may be more common amongst Swedes, or differences in the frequency of the genotype in different populations. So for instance, the *MAOA*-gene particularly the *MAOA-LPR* is from behavioral genetics studies known for interacting with environmental factors to predict behavioral outcomes. A similar gene by environment interaction effect in predicting glioma development may have had differential effects in the two case–control sets depending on sociocultural conditions. Furthermore, since the *MAOA-LPR* is known to influence behavioral outcomes, behavioral differences may also have shaped environmental exposures differently in the two groups. However, no common environmental agents have consistently been associated with glioma risk, apart from the exposure of high dose ionizing radiation, which is a rare event. In our previous studies, we have observed an association with vitamin E, potentially also mediated by the ROS system, but these finding still need independent validation [31].

One important way of further determining whether the tentative general conclusion of a link between development of glioblastoma in males and the rs144551722 is valid will of course be further replication studies. Doing so would be important since an association between rs14551722 and male GBM (as well as a possible association between this disease and *MAOA-LPR*) would have at least three potentially significant implications for glioma research.

### Understanding of the role of monoaminergic pathways in glioma development

As discussed in the introduction there is evidence from previous research to suggest that monoaminergic function might be an important factor in shaping the environment in which gliomas thrive [23–30]. There is also additional evidence to suggest that the MAO-A enzyme may be a key player in regulating these systems [21]. One of the most important aspects of a link between glioma development and *MAO-A*-genotype is as a validation of this line of inquiry in glioma research.

### Strengthening the logic for clinical trials involving MAO-inhibitors

One possibility suggested by the recent pre-clinical study on glioma development is that MAO-inhibiting drugs might possibly become a useful pharmacological adjunct in the treatment of glioma. A finding of a link between *MAO-A*-genotype and development of glioblastoma in the present case–control data sets seems to strengthen the logic in pursuing this possibility further.

### The possibility of MAO-A-genotype as a clinical marker

From a clinical perspective, gliomas share several common features but there are also important individual differences in essential aspects of the disease. That is, there are considerable variations in for instance growth rate, response to therapeutic interventions etc. between individual glioma cases. Although there are some useful molecular markers that may help clinicians make meaningful differentiations between subgroups of gliomas such as *IDH-1* mutation status most such factors remain unknown. Could MAO-A genotype status eventually prove to be a useful clinical marker in glioma cases? Our study does not provide an answer to this question but does suggest that further investigation of this possibility in future studies might be meaningful.

As described above both a strength and a weakness of the present study was that it utilized an evidence-based candidate-gene approach. That is, the study was a direct test of a hypothesis derived from previous research. The advantage is that positive findings made in this way will tend to make biological sense, and that it allows for the discovery of statistically meaningful effects that are not as extreme as those required in theory blind approaches.

However, it should in this context be noted that the rs144551722 has no demonstrated functionality in itself. Instead, it was selected for study because of its close proximity to the *MAOA-LPR* region which is known to be functional [7, 8]. The question whether future replications can validate our findings will of course be important since, experience shows that false positive findings in candidate gene studies have been a common feature in the literature. Such studies would of course also benefit from direct genotyping of the *MAOA-LPR*.

In summary, the results of the present study need additional replication, and a larger sample size, but tentatively suggest the possibility that MAOA-genotype might be associated with glioma development in males. If true, these findings open opportunities for further research concerning glioma tumourigenesis, possible therapeutic effects of MAO-inhibitors, and the possible predictive value of *MAO-A*-genotype as a diagnostic marker in males.
